# The NFL-TBS.40-63 Anti-Glioblastoma Peptide Disrupts Microtubule and Mitochondrial Networks in the T98G Glioma Cell Line

**DOI:** 10.1371/journal.pone.0098473

**Published:** 2014-06-04

**Authors:** Romain Rivalin, Claire Lepinoux-Chambaud, Joël Eyer, Frédérique Savagner

**Affiliations:** 1 Université d'Angers, Angers, France; 2 Laboratoire Neurobiologie & Transgenese, LNBT, UPRES EA-3143, Université d'Angers, Bâtiment IBS-IRIS, Angers, France; 3 CHU Angers, Laboratoire de Biochimie, Angers, France; Columbia University, United States of America

## Abstract

Despite aggressive therapies, including combinations of surgery, radiotherapy and chemotherapy, glioblastoma remains a highly aggressive brain cancer with the worst prognosis of any central nervous system disease. We have previously identified a neurofilament-derived cell-penetrating peptide, NFL-TBS.40-63, that specifically enters by endocytosis in glioblastoma cells, where it induces microtubule destruction and inhibits cell proliferation. Here, we explore the impact of NFL-TBS.40-63 peptide on the mitochondrial network and its functions by using global cell respiration, quantitative PCR analysis of the main actors directing mitochondrial biogenesis, western blot analysis of the oxidative phosphorylation (OXPHOS) subunits and confocal microscopy. We show that the internalized peptide disturbs mitochondrial and microtubule networks, interferes with mitochondrial dynamics and induces a rapid depletion of global cell respiration. This effect may be related to reduced expression of the NRF-1 transcription factor and of specific miRNAs, which may impact mitochondrial biogenesis, in regard to default mitochondrial mobility.

## Introduction

Glioblastoma is a highly aggressive brain cancer that has been designated as grade IV, according to the World Health Organization [1]. It represents an extremely invasive form of glioma and has the worst prognosis of any central nervous system disease. Despite aggressive therapies that include combinations of surgery, radiotherapy and chemotherapy, the median post-diagnostic survival period is approximately one year [2]. Many aspects of glioblastoma contribute to its poor prognosis, including the invasive nature of these abnormal cells [3] and the extreme heterogeneity of this cancer [4]. The lack of specificity for the current treatments and their side effects imply the need to develop new therapeutic strategies that target tumor cells [5].

Microtubule-targeting agents (MTAs) represent an important class of drugs used in the treatment of cancers. Microtubules are ubiquitous cellular polymers composed of heterodimers of α- and β-tubulin subunits [6]. They play major roles in several cellular functions, including intracellular transport, maintenance of cell architecture, cell signaling and mitosis. MTAs exert their anti-tumoral activity by altering microtubule polymerization and dynamics, which causes growth arrest in mitosis and subsequent cell death by apoptosis [7]. Proteins that compose the intermediate filaments are able to bind free unpolymerized tubulin onto specific sites named tubulin-binding sites (TBS) and thus can affect microtubule polymerization *in vitro* and *in vivo* [8]. A peptide derived from the light neurofilament subunit (NFL) that corresponds to the sequence of TBS (NFL-TBS.40-63) can enter specifically into glioblastoma cells by endocytosis [9], where it disrupts the microtubule network and induces cell death by apoptosis [10].

Recent studies have confirmed that interactions between intermediate filaments, notably NFL or Vimentin, and key molecules responsible for the plasticity of the mitochondrial network, including Mitofusin-1 and -2 (MFN1 and 2, respectively) or Dynamin, are necessary to maintain organelle integrity and to allow mitochondrial motility [11]–[13]. The fission process ensures mitochondrial structural quality by removing damaged mitochondria through mitophagy and facilitating apoptosis in conditions of cellular stress [14], [15]. The fusion process could be divided into transient and complete fusion [16]. Contrary to complete fusion, the transient process is essential for promoting mitochondrial metabolism and motility by interplaying with the cytoskeletal anchorage.

A close relationship has been demonstrated between the oxidative phosphorylation (OXPHOS) process and mitochondrial network organization, which is controlled by the balance between fusion and fission events [17]. The failure of mitochondrial fusion–fission dynamics has been involved in the pathogenesis of several neurodegenerative diseases and cancers [18], [19]. Mitochondrial biogenesis is dependant of transcription factors such as nuclear respiratory factors and estrogen-related receptors that coordinate the synthesis of OXPHOS complex subunits encoded by the nuclear and mitochondrial genomes. The transcriptional efficiency of these factors is controlled by coactivators from the peroxisome proliferator-activated receptor γ coactivator-1 (PGC-1) family, i.e., PGC-1α, PGC-1β and the PGC-1-related coactivator (PRC), that integrates mitochondrial biogenesis and function to various environmental signals [20]. We previously showed that the ubiquitous PRC member was able to control mitochondrial fission by modulating the Fission-1 (FIS1) expression level in cancer cells, in addition to its effect on mitochondrial biogenesis [21].

Numerous studies have highlighted selected miRNAs related to glioma pathogenesis [22]. Some of them have potential applications as novel diagnostic and prognostic indicators. Thus, the re-expression of miR-34a encoded at Chr1p36.22, a region deleted in many glioblastomas, could be associated with reduced tumor proliferation, cell migration and invasion [23]. Conversely, miR-21 has been identified as an anti-apoptotic factor and presents a significant up-regulation in glioblastoma, while its inhibition induced apoptosis in glioblastoma cells *in vitro* and *in vivo* [24], [25]. This miRNA is involved in the down regulation of the tumor suppressor gene PTEN, in caspase 3/7 activation and confers a drug resistance to cancer cells [26]. Moreover, an over-expression of miR-221 has been linked with increased cellular proliferation and an over-expression of the *c*-KIT gene [27], [28]. These miRNAs have also recently been related to a pool of miRNAs called mitomiRs, which are associated with the mitochondrial compartment [29]. Their role in the control of mitochondrial functions and cell redox status is now established [30], [31].

In this study, we focused on the role for MTAs in the OXPHOS process and the dynamics of mitochondrial networks. For this purpose, we used the T98G cellular model of human glioblastoma, in which we have previously demonstrated the incorporation and cytoskeleton effect for 10 µM NFL-TBS.40-63 peptide [8]–[10], [32].

## Materials and Methods

### Cell lines and peptide design

Human T98G glioblastoma cells and mouse NIH-3T3 embryonic fibroblast cells were obtained from ATCC (Manassas, VA, USA). They were grown in T75 flasks at 37 °C under 5% CO2, in Dulbecco's modified Eagle's medium with 1 g/l glucose, which was supplemented with 5% L-glutamine, 10% fetal calf serum (both from Lonza, Walkersville, MD, USA) and 5% (penicillin/streptomycin (Sigma, Saint-Quentin Fallavier, France). A biotinylated peptide corresponding to NFL (NFL-TBS.40-63: YSSYSAPVSSSLSVRRSYSSSSGS) and a similarly labeled scrambled peptide (NFL-SCR: SLGSPSSSVRASYSSSRSYVYSSS) were synthesized (with more than 95% purity) by MilleGen (Toulouse, France) and dissolved in water at 1 mM concentration. They were used at final concentrations of 2, 5 and 10 µM for 6 h at 37 °C to evaluate their cellular effects.

### Quantitative mRNA and miRNA analyses

T98G cells were tested for peptide effects on independent triplicate cell cultures. Cells were harvested following a trypsin-EDTA 1× (Sigma) treatment for 5–10 min and centrifugation. Total RNA was extracted from cells pellets using the RNeasy® Mini Kit (Qiagen, Valencia, CA, USA) according to the manufacturer's recommendations. Reverse transcription was performed on 1 µg of total RNA with the Advantage RT-for-PCR kit (Clontech, Palo Alto, CA, USA), following the manufacturer's recommendations. Real-time quantification was performed on a 96-well plate using the Power SYBR® Green Master mix and the ABI 7900 apparatus (Applied Biosystems by Life technologies, Grand Island, NY, USA). Ten genes were tested for quantitative expression: MFN2 and FIS1 for the mitochondrial fusion and fission process, PRC/PPRC1, PGC-1α/PPARGCA1, NRF-1, CYCS (Cytochrome c) and TFAM (Mitochondrial transcription factor A) for mitochondrial biogenesis and its functions, PTEN, NAIP and FGFR3 as genes directly targeted by miR-21, 221 and 100, respectively [33], [34]. The data were normalized to β-globin, and the relative expression level of specific mRNA was calculated with the usual 2–ΔΔCt method. The sequences of primers used in this study are referenced in Table 1.

**Table 1:** Primers used for mRNA and miRNA quantification.

PRC/PPRC1	For: 5′-CACTGGTTGACCCTGTTCCT-3′ Rev: 5′-GTGTTTCAGGGCTTCTCTGC-3′	miR-125a-3p	5′-GGTGAGGTTCTTGGGAGCC-3'
CYCS	For: 5′-CCAGTGCCACACCGTTGAA-3′ Rev: 5′-TCCCCAGATGATGCCTTTGTT-3′	miR-181b	5′-ATTCATTGTTGTCGGTGGGT-3'
PGC1α/PPARGCA1	For: 5'-ACTCAAGTGGTGCAGTGACC-3' Rev: 5'-CTGGGTACTGAGACCACTGC-3'	miR-107	5′-GTCGTGAGCAGCATTGTACAG-3'
NRF-1	For: 5'-GGAGTGATGTCCGCACAGAA-3' Rev: 5'-CGCTGTTAAGCGCCATAGTG-3'	miR-30a	5′-TGTAAACATCCTCGACTGGAAG-3'
TFAM	For: 5'-CCGAGGTGGTTTTCATCTGT-3' Rev: 5'-CAGGAAGTTCCCTCCAACGC -3'	miR-146b	5′-TGAGAACTGAATTCCATAGGCT-3'
FIS1	For: 5'- GGAGGAACAGCGGGATTACGT-3' Rev: 5'- CTTCATGGCCTTGTCAATGAGC-3'	miR-96	5′-TTTGGCACTAGCACATTTTTGCT-3'
MFN2	For: 5'- GAAGAACAGGTTCTGGACGTC-3' Rev: 5'- CCTCATGGCCATCTGTGCCC-3'	miR-221	5′-ACCTGGCATACAATGTAGATT-3'
PTEN	For:5′- CGGCAGCATCAAATGTTTCAG-3′ Rev: 5′-AACTGGCAGGTAGAAGGCAAC-3′	miR-885	5′-AGGCAGCGGGGTGTAGTGGATA-3'
NAIP	For: 5′-TAGACTTGCGTCCTTCAGGAA-3′ Rev: 5′- CTGCAACTCCCACAGCTGATT-3′	miR-218	5′-CGTTGTGCTTGATCTAACCATGT-3'
FGFR3	For: 5′-ACCTTCAAGCAGCTGGTGGA-3′ Rev: 5′-CTAGGGACCCCTCACATTGT-3′	miR-100	5′-GCCCAAGCTTGTATCTATAGGTAT-3'
β-Globin	For: 5′-CAACTTCATCCACGTTCACC-3′ Rev: 5′-ACACAACTGTGTTCACTAG-3′	miR-31	5′-AGGCAAGATGCTGGCATAGCT-3'
U5snRNA	5′-AAATTGGAACGATACAGAGAAG-3′	miR-21	5′-CGGTAGCTTATCAGACTGATGTTG-3'

For miRNA analyses, cDNA was first produced using the VILO miRNA cDNA Synthesis Kit (Invitrogen, Carlsbad, CA, USA) according to the manufacturer's protocol. Using Express SYBR GreenER qPCR SuperMix (Invitrogen), real-time PCR was carried out on a ABI 7900 apparatus (Applied Biosystems by Life technologies) using a universal primer and forward primers specific to each miRNA, according to the NCode miRNA Database (Table 1). For each sample, three independent reverse transcription reactions were performed, and each reaction was assayed in duplicate by real-time PCR. MiRNA levels were normalized to U5 snRNA, a snoRNA (small nucleolar RNA), which has been established as the most stably expressed reference gene [35].

### Western blot analyses

T98G and NIH-3T3 cells were incubated with 10 µM of NFL-TBS.40-63 or NFL-SCR. After 6 hours of treatment, the cells were rinsed in phosphate-buffered saline 1X (PBS 1X; Sigma-Aldrich, St. Louis, MO, USA), trypsinized and collected in centrifuge tubes. The cells were lyzed and the protein concentrations were measured using a protein assay (Thermo Scientific, Waltham, MA, USA). 20 µg of the proteins were separated by SDS-PAGE, transferred to poly (vinylidene difluoride) membranes (Hybond-P, Amersham, Buckinghamshire, UK) and incubated either with dilutions of the following monoclonal antibodies: 1/10000 anti-α−tubulin (Abcam, Cambridge, UK), 1/2000 anti-complex-IV, subunit IV (COX4, MS408, Mitosciences, Eugene, OR, USA) and 1/2000 anti-complex-II, subunit Ip (SDHB, MS203, Mitosciences), or with dilutions of the following polyclonal antibodies (All from Abcam): 1/1000 anti β-actin (Ab-8229), 1/5000 anti NRF-1 (Ab-86516) and 1/2000 anti-PGC-1α (Ab-54481). After several washes in TBS-Tween (with 0.1% Tween-20), the membranes were incubated with an appropriate chemiluminescent-labeled, horseradish peroxidase-conjugated secondary antibody (Jackson ImmunoResearch, WestGrove, PA, USA). The blots were developed using the enhanced chemiluminescence method (ECLplus, Amersham). Signal quantification was performed via non-saturating picture scanning using a Gel Doc 1000 Molecular Analyst apparatus (Biorad, Hercules, CA, USA).

### Mitochondrial oxygen consumption analyses

NIH-3T3 and T98G cells were seeded in Seahorse XF-24 plates (from Seahorse Bioscience, North Billerica, MA, USA). The cells were tested with 2, 5, or 10 µM of NFL-TBS and compared to 10 µM NFL-SCR. After 6 h of treatment, the cells were changed to an unbuffered DMEM (DMEM base medium supplemented with 17.5 mM glucose, 1 mM sodium pyruvate, 31 mM NaCl and 2 mM Glutamine, pH 7.4) and incubated at 37 °C in a non-CO2 incubator for 1 h. Three baseline measurements of oxygen consumption rate were collected on the XF 24 Seahorse apparatus using the XF cell Mito Stress kit (#101706 from Seahorse Bioscience). After the Seahorse analysis, the cells were lyzed, and the protein concentration was measured using a protein assay (Thermo Scientific, Waltham, MA, USA). The oxygen consumption of each well was normalized according to the total protein amount (pmol/min/µg).

### Confocal microscopy

To study the molecular impact of the NFL-TBS.40-63 peptide on T98G cells, we first used mitochondria (Mitotracker Red CMX ROS) and microtubule markers (Alexa647-labeled anti-tubulin antibody, both from Life Technologies, Carlsbad, CA, USA) on fixed cells. The cells were seeded in 24 well plates (2×104 cells/well) containing coverslips. After 48 h, the cells were incubated in 10 µM biotinylated peptides (NFL-TBS.40-63 or NFL-SCR) for 6 h at 37°C. After washing with the media, the cells were incubated for 15 min at 37 °C with 100 nM mitotracker Red CMX ROS diluted in the media. After washing with PBS, the cells were fixed in 4% paraformaldehyde in PBS for 10 min. Following three washes in PBS, the cells were incubated for 10 min in a permeabilization solution (Pipes 0.1 M, EGTA 1 mM, MgCl2 0.1 M, 4% PEG 8000, 0.5% triton X-100, in PBS, pH 7.4). They were washed three times in PBS before incubation in a blocking solution (5% bovine serum albumin) for 15 min. Permeabilized cells were incubated overnight with a mouse anti-α tubulin antibody (Sigma) at 1/500. Then, tubulin and biotinylated peptides were localized using Alexa 647 nm anti-mouse antibody and streptavidin Alexa 488 nm (Life Technologies) respectively, at 1/200 for 1 h. After washing by PBS, cell nuclei were stained by 3 µM 4,6-diamidino-2-phenylindole (DAPI, Sigma) for 5 min. Finally, coverslips were mounted with an anti-fading mounting medium (Prolong, Life Technologies) and observed with a confocal Nikon A1RSI instrument. The images were analyzed using Nikon NIS-element software.

To study dynamics of the mitochondrial network, we have realized live cell imaging after 15 hours of cell treatment by 10 µM FITC-NFL-TBS.40-63 or FITC-scrambled peptide in Labtek four-chambered coverglasses (Nalge Nunc International), followed by 10 µM mitotracker Red CMX Ros treatment during 15 minutes. After washing in PBS, cells were rapidly placed in culture media according to recommended protocol [36]. Mitochondrial imaging was acquired within 3 minutes with 5 seconds intervals using confocal Nikon A1RSI instrument. Minimum laser power was used to minimize photo bleaching. Image analysis was done using the tracking suite of Metamorph software application module (Molecular Devices, Sunnylade, CA, USA).

To determine the migration ability of T98G cells treated by NFL-TBS 40-63 peptide or scramble, we have used a transwell migration assay as previously described [10].

### Statistical analyses

All experiments were repeated at least three times. The data are represented as mean values ± standard deviation (SD), with N representing the number of experiments. The statistical significance of the variations observed was assessed using the Mann-Whitney test. The differences were considered significant at *P*<0.05. All analyses were performed using Prism3.00 (GraphPad software, San Diego, CA).

## Results

### The NFL-TBS.40-63 peptide regulates the number of mitochondria and their function in human glioblastoma T98G cell line

**Figure 1:**
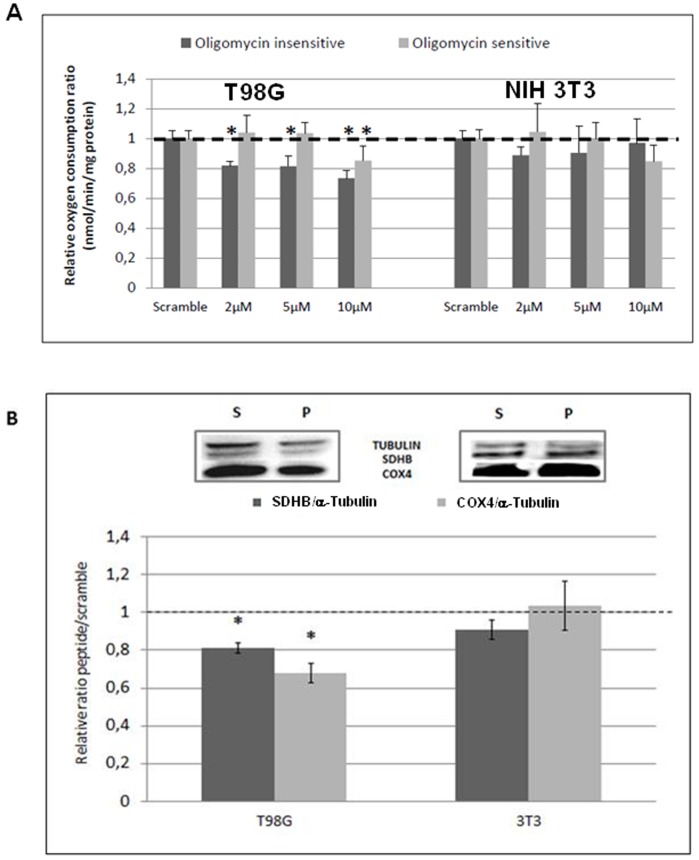
Action of the NFL-TBS.40-63 peptide on mitochondrial number and functions in T98G cells and NIH 3T3 control cells. 1A: The relative oxygen consumption was measured using a mitostress kit and a Seahorse XF-24 apparatus (from Seahorse Bioscience, North Billerica, MA, USA). The oligomycin-insensitive fraction represents non-phosphorylating respiration, which was recorded after the inhibition of ATP synthase with oligomycin. The oligomycin-sensitive fraction represents the phosphorylating respiration, i.e., the fraction used for ATP synthesis. Results are expressed relative to oxygen consumption of scramble treated cells used as control (pmol/min/mg protein). 1B: The protein expression of mitochondrial subunit IV of complex IV (COX4, MS408, Mitosciences) and subunit Ip of complex II (SDHB, MS203, Mitosciences) were measured by Western blot analysis after a 6-hour exposure to 10 µM NFL-TBS.40-63 peptide and normalized to the α-tubulin level (65 KDa; Abcam, Cambridge, UK). The protein expression for the peptide-treated samples was expressed relative to that of the scramble-treated samples. S: Scramble; P: NFL-TBS.40-63 peptide. Results are expressed relative to protein expression ratio of scramble treated cells used as control. The values represent the average ± SD for three separate determinations (*N* = 3). *: *P*<0.05 versus control.

We investigated the impact of a 6 hours-incubation in various NFL-TBS.40-63 peptide concentrations (2 to 10 µM) on mitochondrial respiratory function and biogenesis from T98G cells and NIH3T3 control cells [8], [9]. A mitostress test was performed to investigate the main parameters of the OXPHOS process, including basal respiration rate, ATP turnover, proton leak and maximal oxygen consumption rate. Mitochondrial respiration is divided into two fractions. The oligomycin insensitive fraction corresponds to non-phosphorylating respiration and is recorded after the inhibition of ATP synthase with 1 µM oligomycin. The oligomycin-sensitive fraction represents the phosphorylating respiration and is the fraction used for ATP synthesis, which is calculated by subtracting the nonphosphorylating respiration rate from the basal respiration rate. The NFL-TBS.40-63 peptide significantly affected both oligomycin-sensitive and insensitive fractions at 10 µM (Figure 1A). The global oxygen consumption rate was reduced at all concentrations, with specific and significant decreases in the oligomycin-insensitive fractions of approximately 20% at the 2 µM and 5 µM concentrations. However, no defect in the fraction used for ATP synthesis (oligomycin-sensitive fraction) could be noticed at these lowest concentrations, contrary to that observed for 10 µM peptide. Conversely, for the NIH-3T3 cells, none of the two oligomycin fractions seemed to be significantly affected by the peptide even if a tendency to decrease could be noticed at 10 µM peptide for the oligomycin-sensitive fraction. To explore the impact of 10 µM NFL-TBS.40-63 treatment on mitochondrial biogenesis, we have investigated the protein expression level of mitochondrial complex subunits using Western blot analysis (Figure 1B). Our results show a significant reduction in SDHB (complex II subunit) by 30% and of COX4 (complex IV subunit) by 20%, respectively, in T98G cells, when compared to the scrambled peptide. In NIH-3T3 cells, no such reduction was observed, in accordance with our previous results for non-permeant NIH-3T3 cells for this peptide [8]. These results indicate a primary impact of the peptide on mitochondrial functions at low concentrations (2 and 5 µM), which had no detectable effect on the microtubule network. However, at 10 µM of peptide, both networks were affected [8].

### The NFL-TBS.40-63 peptide rapidly affects the regulatory factors of the mitochondrial biogenesis in the T98G cell line

**Figure 2:**
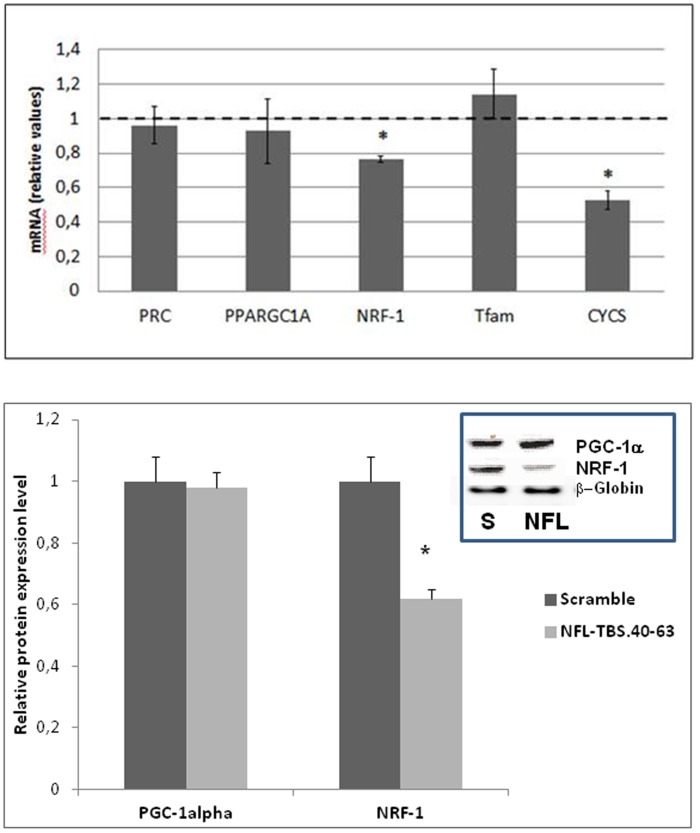
Expression analysis of the genes involved in the control of mitochondrial biogenesis in T98G cells treated by 10 µM of NFL-TBS.40-63 peptide. 2A: Quantitative PCR analysis: PRC (PPRC1) and PGC-1α (PPARGC1A) coactivators, NRF-1 transcription factor, mitochondrial transcription factor TFAM and a component of the respiratory chain, Cytochrome c (CYCS), were measured. The data are expressed in relative units (mRNA expression of a specific gene normalized to β-globin mRNA expression) and expressed relative to the control, which was assigned a unit value. The values are the average ± SD for three separate determinations (*N* = 3). *: *P*<0.05 versus control. 2B: Western blot analysis: The protein expression of PGC-1α (105KDa, Ab- 54481, Abcam) and NRF-1 (54 KDa, Ab-86516, Abcam) were measured by Western blot analysis after a 6-hours exposure to 10 µM NFL-TBS.40-63 peptide and normalized to the β-actin level (45 KDa; Abcam). Results are expressed relative to protein expression ratio of scramble treated cells which was assigned a unit value. The values are the average ± SD (*N* = 3). *: *P*<0.05 versus control.

The expression of five essential genes that control mitochondrial biogenesis and function -PRC (PPRC1) and PGC-1α (PPARGC1A) coactivators, NRF-1 transcription factor, mitochondrial transcription factor TFAM and a component of the respiratory chain (CYCS) - were investigated in T98G cells. We observed that reduced mitochondrial oxygen consumption at 10 µM NFL-TBS.40-63 peptide was associated with a significantly reduced expression of NRF-1 and its target gene CYCS by 20% and 50%, respectively (Figure 2A). However, no significant changes in the expression levels of PRC, PGC-1α or TFAM could be observed after 6 hours of NFL-TBS.40-63 treatment. These results indicate that NFL-TBS.40-63 peptide has a rapid impact on the transcriptional machinery regulating the mitochondrial biogenesis. This regulation is independent of the expression level for PRC and PGC-1α and could be related to the post-transcriptional regulation of NRF-1 expression.

We confirmed, at the protein level, the decrease in NRF-1 expression compared to PGC-1α following the 10 µM peptide treatment (Figure 2B).

### The NFL-TBS.40-63 peptide alters microtubules and mitochondrial organization at 10 µM Concentration

**Figure 3:**
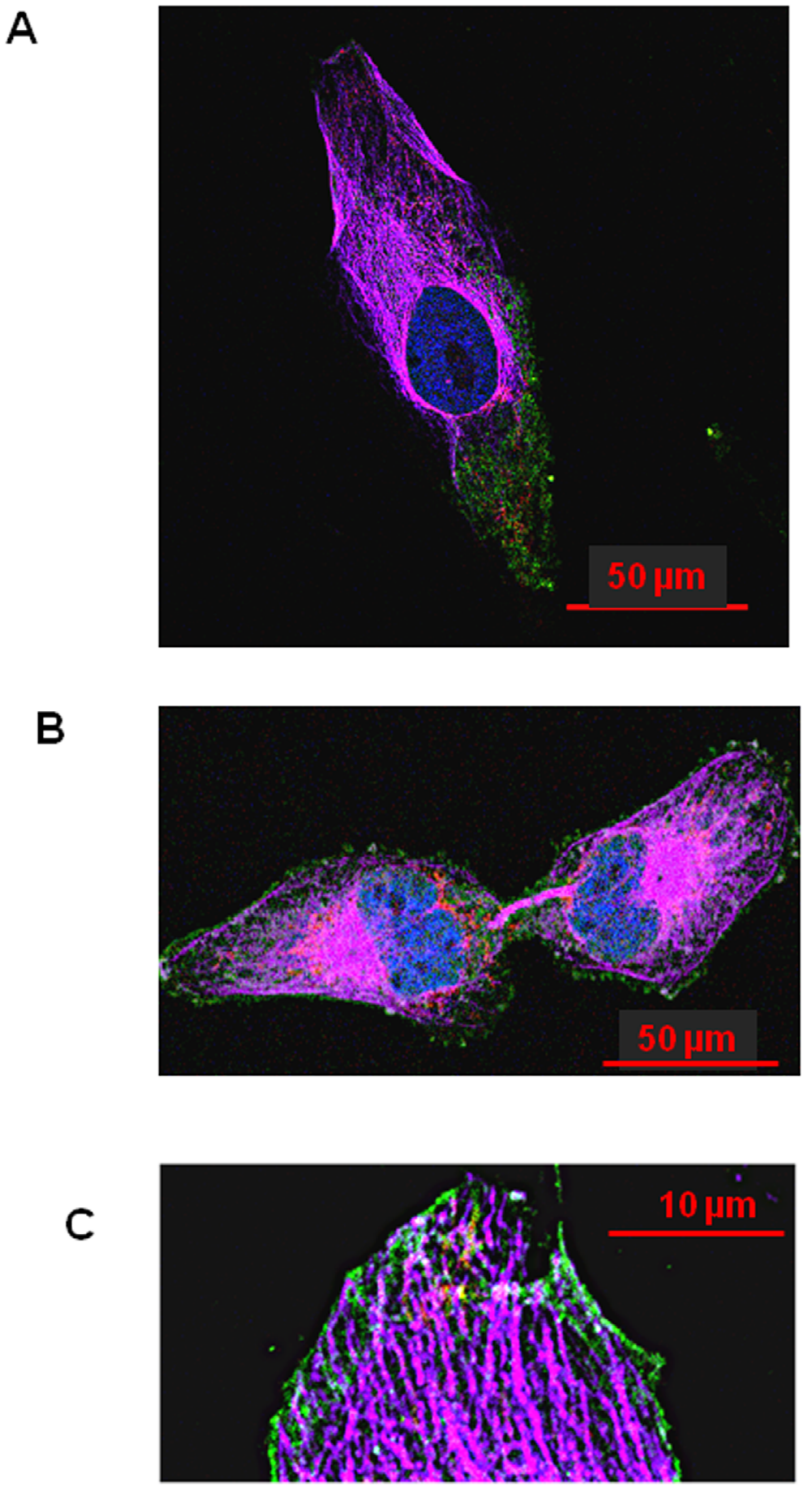
Effects of 10 µM of NFL-TBS.40-63 peptide on mitochondrial and microtubule networks in human T98G glioblastoma cells. 3A: NFL peptide accumulates within the cell in a polarized manner, limiting the density of the microtubules and mitochondrial networks. 3B: NFL peptide accumulates at the basis of the midbody and excludes the microtubule network. 3C: The peptide surrounds the microtubules' tips and limits filopodia formation. Microtubules were detected using an Alexa647-labeled, anti-α-tubulin antibody (purple); biotinylated NFL-TBS.40-63 peptide was labeled with streptavidin Alexa488 (green), the nuclei with diamidino phénylindole (DAPI; blue) and mitochondria with a mitotracker (RedCMX ROS). The cells were examined with a Nikon A1RSI confocal microscope and the images were analyzed with Nikon NIS-element software. The *red bars* are the measuring scale.

**Figure 4:**
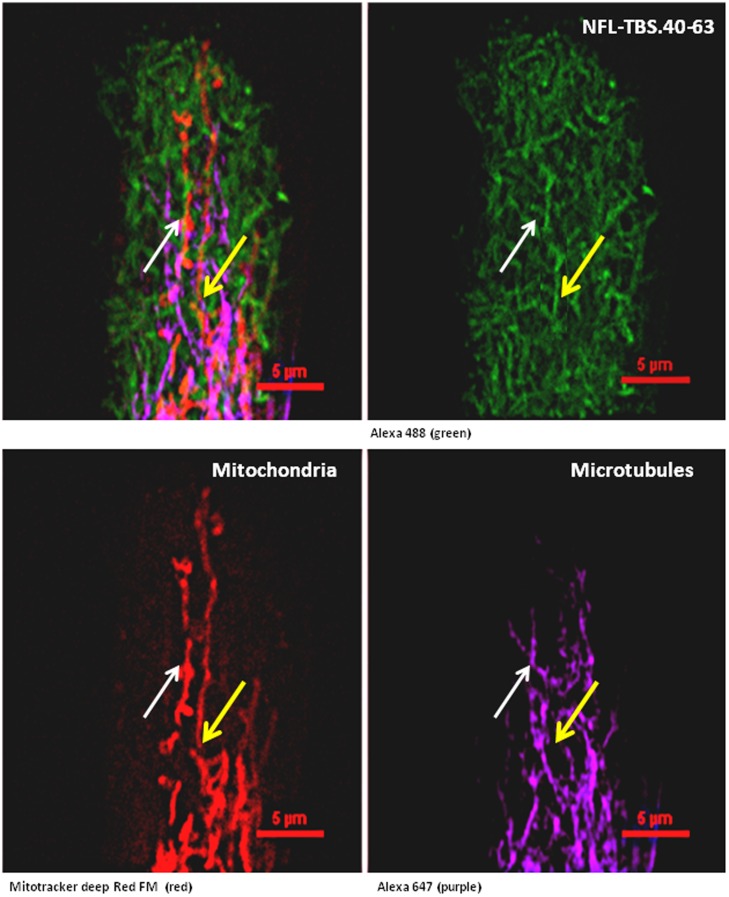
The NFL-TBS.40-63 peptide reorganizes mitochondrial networks in human T98G glioblastoma cells. The white arrow indicates a mitochondrial network (bottom left) superposed with a microtubule network (bottom right). The yellow arrow indicates a mitochondrial network (bottom left) superposed with long peptide sequences (top right). The microtubules were detected using an Alexa647-labeled, anti-α-tubulin antibody (purple); biotinylated NFL-TBS.40-63 peptide was labeled with streptavidin Alexa488 (green), the nuclei with diamidino ph?nylindole (DAPI; blue) and the mitochondria with a mitotracker Red CMX ROS). The cells were examined with a Nikon A1RSI confocal microscope and the images were analyzed with Nikon NIS-element software. The red bars are the measuring scale.

Previously, we have shown that T98G human glioblastoma cells internalized the NFL-TBS.40-63 peptide at a 10 µM concentration, which induces the disruption of their microtubule network [8]. Consequently, tubulin is aggregated around the nucleus, while cells lose their extensions and become spherical. Using markers of both mitochondrial and microtubule networks, in association with a marked peptide, confocal microscopy showed that the peptide entered in T98G and accumulated in a polarized manner (Figure 3A and Figure S1 in File S1 for unmerged images). This was related with a reduction in microtubule and mitochondrial density where the peptide accumulated. It was also convincing for dividing cells where the basis of the midbody was enriched with peptide and mitochondria but completely devoid of microtubule (Figure 3B). The NFL-TBS.40-63 peptide was also able to surround microtubule tips and should limit filipodia formation (Figure 3C), in accordance with our previous results showing the reduction of cell motility at a low peptide concentration [10]. We observed that the peptide was able to co-localize with both the microtubules and the mitochondria to modify the architecture of their networks contrary to that observed in untreated cells (Figure 4 and Figure S4 in File S1). We also observed that mitochondria could be structured independently of the microtubule network with co-localized mitochondrial network and long chains of NFL-TBS.40-63 peptide sequences (Figure 4, yellow arrows).

### The NFL-TBS.40-63 peptide reduces mitochondrial dynamics

**Figure 5:**
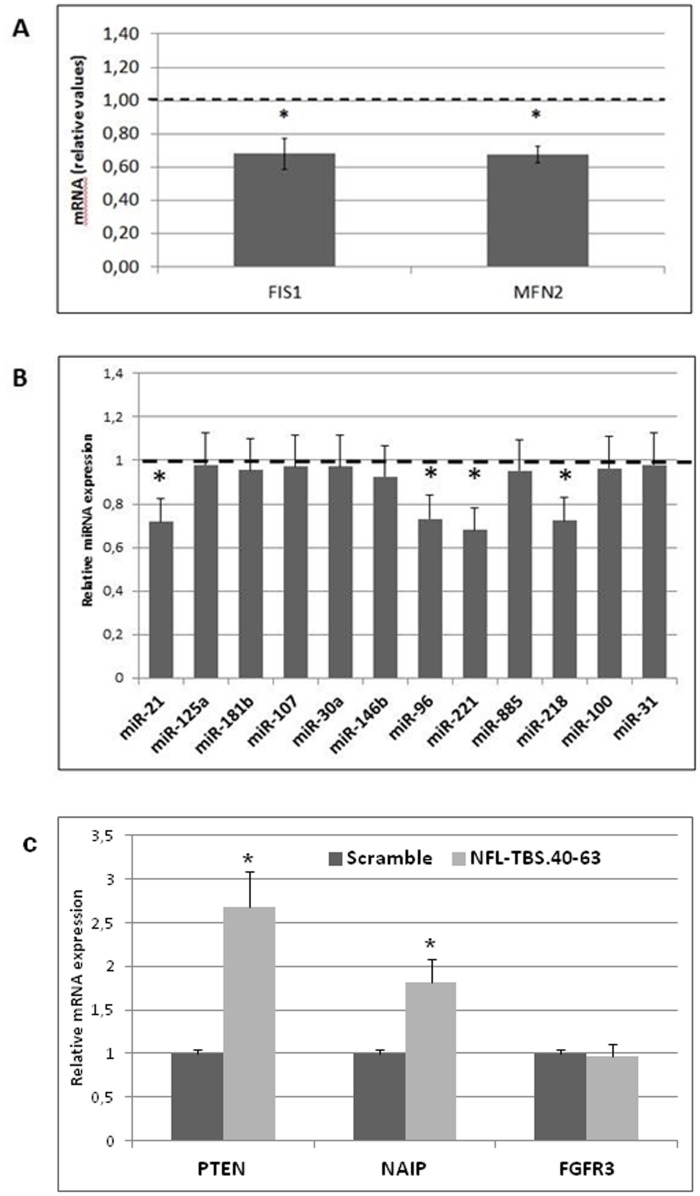
Quantitative PCR analysis of mitochondrial fission/fusion actors and relevant differentially-expressed miRNA-mRNA complexes in human T98G glioblastoma cells. 5A: Quantitative PCR analysis of mitochondrial fission/fusion actors (FIS1 and MFN2) in T98G cells. The data are expressed in units (mRNA expression of a specific gene normalized to β-globin mRNA expression) that are relative to the control, which was assigned a unit value. 5B: Quantitative PCR analysis of relevant differentially expressed miRNA in T98G cells. The data are expressed in units (miRNA expression relative to U5 snRNA) that are relative to the control, which was assigned a unit value. 5C: Quantitative PCR analysis of PTEN and NAIP mRNA, directly targeted by miR-21 and miR-221, respectively. FGFR3 expression was used as negative control of miR-100, which expression level was unchanged by peptide treatment. The data are expressed in units (mRNA expression of a specific gene normalized to β-globin mRNA expression) that are relative to the control, which was assigned a unit value. The values are the average ± SD for three separate determinations (*N* = 3). *: *P*<0.05 versus control.

We explored the impact of NFL-TBS.40-63 on the mitochondrial fission–fusion balance using the master regulator of mitochondrial dynamics, MFN2, which is responsible for mitochondrial multiplication and FIS1, which is involved in mitochondrial fission (Figure 5A). Here, we showed that, even if both factors presented a significant decrease in expression level, the FIS1/MFN2 ratio, which refers to the balance between dynamic events, was conserved. Rather, this conserved modeling balance involved differences mainly in mitochondrial motility resulting from abnormal cytoskeletal anchorage. Thus, we have observed a decrease in mitochondrial motility (mean speed motility 7-times slower) using mitochondrial network imaging as well as decrease in cell motility (53% mean decrease in cell migration) using the transwell assay, in peptide-treated cells compared to scramble-treated (Figures S2 and S3 in File S1, respectively).

### The NFL-TBS.40-63 peptide induces post-transcriptional regulation

We have analyzed the expression of relevant miRNAs associated with mitochondria, drug resistance, or both and found that a 6-hours treatment with 10 µM of NFL-TBS.40-63 peptide induced a significant change in expression for four miRNA: miR-96, miR-218, miR-21 and miR-221 (Figure 5B). Two of these miRNA (miR-21 and miR-221) were found to be overexpressed in many cancer cells, including glioblastoma, as they had anti-apoptotic and anti-mitophagic effects. We have confirmed the effect of miR-21 and miR-221 by measuring up regulation of PTEN and NAIP genes, directly targeted by these miRNA, compared to unchanged expression level for FGFR3 gene targeted by miR-100 (Figure 5C).

## Discussion

A close interaction between mitochondria and the cytoskeleton is essential to ensure the proper distribution of mitochondria within a cell. Recent studies have highlighted interactions between intermediate filaments, notably NFL or Vimentin and the key molecules necessary for the maintenance of organelle integrity and mitochondrial motility [11]–[13]. The NFL-TBS.40-63 peptide is able to alter microtubule formation when it is internalized by T98G glioblastoma cells and inhibits their proliferation [8]. In this study, we have evaluated the effect of NFL-TBS.40-63 peptide internalization on mitochondrial biogenesis and function.

Our observations revealed a negative impact on T98G cell respiration after 6 hours of NFL-TBS.40-63 peptide treatment (2 to 10 µM). This action on mitochondrial function, at lower concentrations than those necessary to disturb the cytoskeleton (10 µM), could be related to a primary modification of the mitochondrial motility. It has been shown that peptides derived from the N-terminal domain of intermediate filaments, like desmin, vimentin and keratin, can interact with the unpolymerized tubulin [8]. A recent study demonstrated that the N-terminal domain of vimentin (residues 41–94) can also directly bind mitochondria and serve as an adaptor between actin microfilaments and mitochondria [13]. We suggest that the primary action of NFL-TBS.40-63 leads to sequential organization of the peptide, which disturbs the cytoskeleton and reorganizes the mitochondrial network. This should be related to the conserved FIS1/MFN2 ratio we observed at higher peptide concentrations. At a 10 µM peptide treatment, we also revealed a colocalization of NFL-TBS.40-63 with mitochondria and a specific accumulation at the microtubules' extremities, which may limit membrane ruffling, as previously reported [37], [38].

This study revealed that the NFL-TBS.40-63 peptide provokes a redistribution of mitochondria throughout the cytoplasm. Mitochondria were able to reorganize along the peptide from end to end, in order to form a polarized but less dense network and reduce cell respiration. Mitochondria and autophagy are linked to homeostatic elements that act in response to changes in the cellular environment, such as energy, nutrients and stress. Thus, defects in plasticity could simultaneously impair autophagy, which may result in increased risk for various human diseases [39], [40]. The peptide treatment induces an inhibition of FIS1 and MFN2 gene expression. As has been shown, deregulation of mitochondrial fusion or fission is associated with alterations in the organization of the mitochondrial network and with the inhibition of energy metabolism [41]. Alterations in energetic metabolism cause defects in respiratory chain subunits and may lead to mitochondrial network fragmentation [42]. Western blotting analyses indicated that decreases in the OXPHOS process were also related to a decrease in mitochondrial biogenesis when using 10 µM of NFLTBS.40-63 peptide, regarding protein levels for two subunits of the respiratory chain complexes (SDHB and COX4) and for transcription factor NRF-1. This rapid reduction of mitochondria after 6 hours of peptide treatment may be related to the induction of mitophagy. Thus, the PGC-1α/PRC pathway, which is related to the transcriptional regulation of mitochondrial biogenesis, was not affected after 6 hours of treatment, while NRF-1 and CYCS were repressed; this suggests a lack of extra-cellular signal regulation or a delayed PGC-adaptive response to energy depletion. Moreover, this could suggest a rapid regulation of mitophagy/biogenesis balance through post-transcriptional pathways, as recently reported [43], [44].

We found that the expression of two relevant miRNAs-miR-21 and miR-221-was altered by a 6-hours treatment with the NFL-TBS.40-63 peptide, compared to the scrambled control. These miRNAs are referred to as oncomirs, as they have anti-apoptotic and proliferative effects [25], [26], [28], [45]. In human tumors, miR-21 down-regulates the expression of PTEN, which is involved in mitophagy through its negative regulation of PINK1 (PTEN-induced kinase 1) [34]. Up-regulation of miR-221 has also been correlated with down-regulation of one of its targets, NAIP (NLR-family, apoptosis inhibitory protein), which is involved in neurodegeneration and apoptosis regulation [33]. On the contrary, over-expression of miR-218 and miR-96 were associated with apoptosis induction through targeting the PINK1/NF-KB pathway and FOXO1, respectively [46], [47]. The inverse functions of these miRNAs on apoptosis or mitophagy should be considered, depending on their half-time. Thus, miR-21 is considered to be one of the most long-lived miRNAs [48].

In conclusion, we showed that a 6-hours treatment with 10 µM of NFL-TBS.40-63 peptide induces a direct reduction in the global respiration of cells that have internalized the peptide. This effect can be related to a reduction of the main transcription factor involved in mitochondrial biogenesis and in the OXPHOS process. The treatment is efficient in reducing the expression of miRNAs that are involved in glioblastoma pathogenesis and MTA resistance. To our knowledge, this is the first report showing the role played by this peptide in the regulation of global respiration for glioblastoma cells. Our results show that disrupting a microtubule network affects the mitochondrial network distribution, thus confirming the close relationship between these two networks.

## Supporting Information

File S1 (PDF)
