# Supplementary material for: The NFL-TBS.40-63 Anti-Glioblastoma Peptide Disrupts Microtubule and Mitochondrial Networks in the T98G Glioma Cell Line
Source: PLoS One. 2014 Jun 4;9(6):e98473. doi: 10.1371/journal.pone.0098473 (PMC4045719; doi:10.1371/journal.pone.0098473)

Figure S1 : Unmerged images from Fig. 3

Microtubules were detected using an Alexa647-labeled, anti-α-tubulin antibody (purple); biotinylated NFL-TBS.40-63 peptide was labeled with streptavidin Alexa488 (green), the nuclei with diamidino phénylindole (DAPI; blue) and mitochondria with a mitotracker (RedCMX ROS). The *red bars* are the measuring scale.

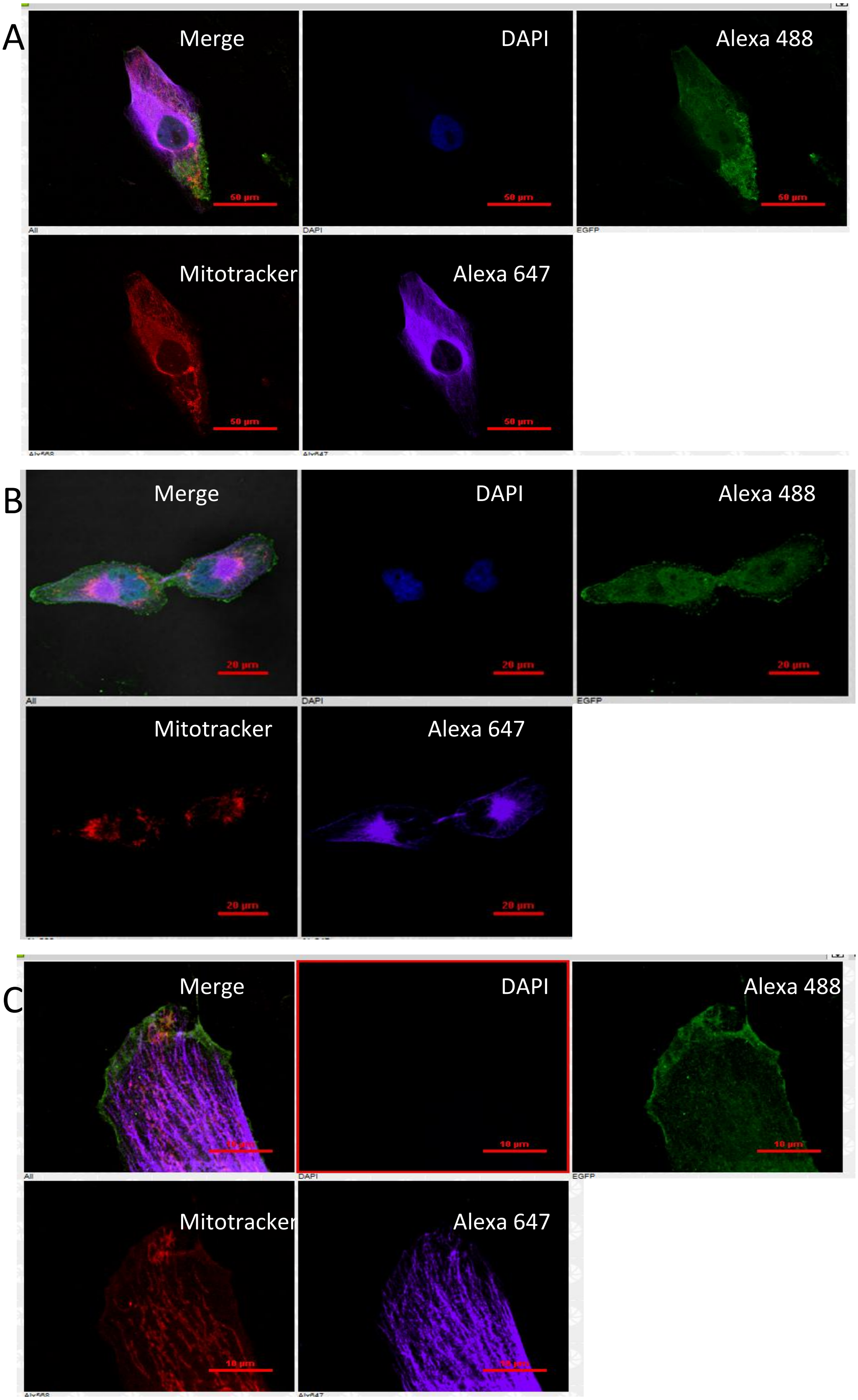

Figure S2 : In vitro effect on mitochondrial motility of 15 hours exposure with 10 µM NFL-TBS.40-63 peptide compared to scramble, using live confocal microscopy and Metamorph software (*N=6*). Time duration 3 minutes, image every 5 seconds. T30 and T60 in seconds

Scramble

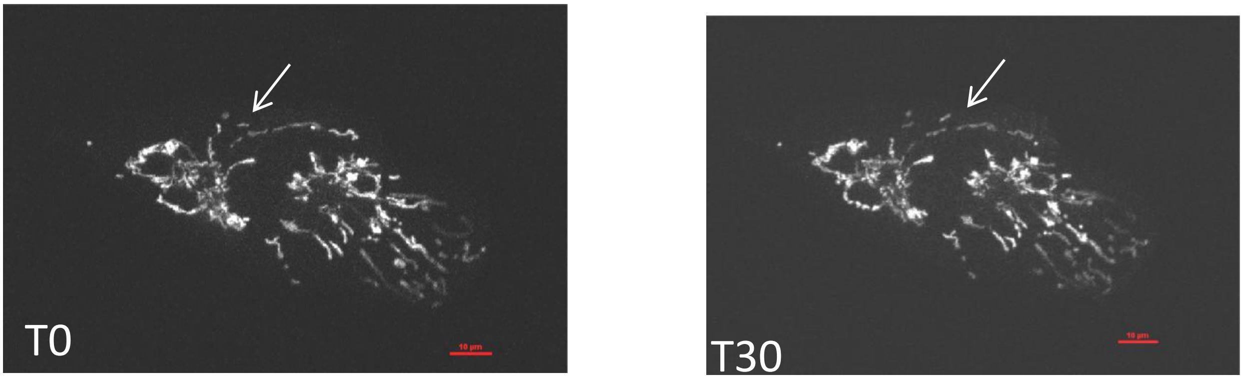

Peptide

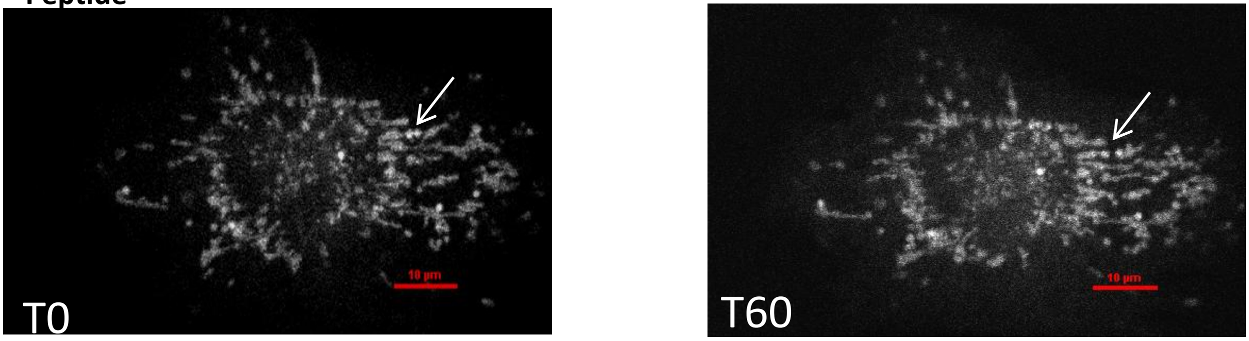

Figure S3 : In vitro effect on T98G cell migration of 15 hours exposure with 10 µM NFL-TBS.40-63 peptide, using Transwell migration assay . (* *P*< 0.05)

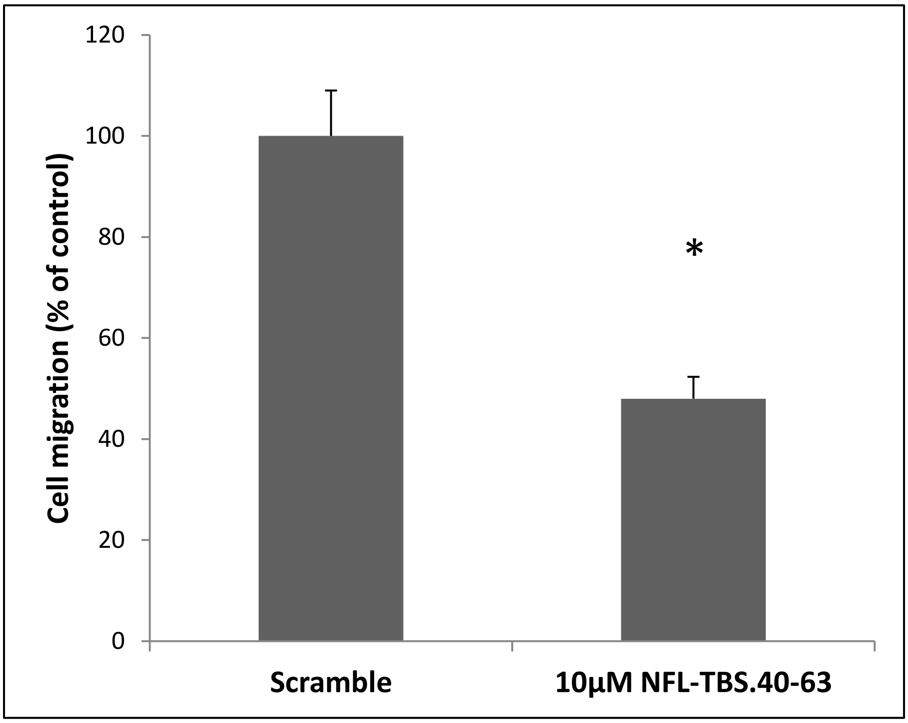

Figure S4: Confocal imaging of untreated T98G cells

Microtubules were detected using an Alexa647-labeled, anti-α-tubulin antibody (purple); biotinylated NFL-TBS.40-63 peptide was labeled with streptavidin Alexa488 (green), the nuclei with diamidino phénylindole (DAPI; blue) and mitochondria with a mitotracker (RedCMX ROS). The *red bars* are the measuring scale.

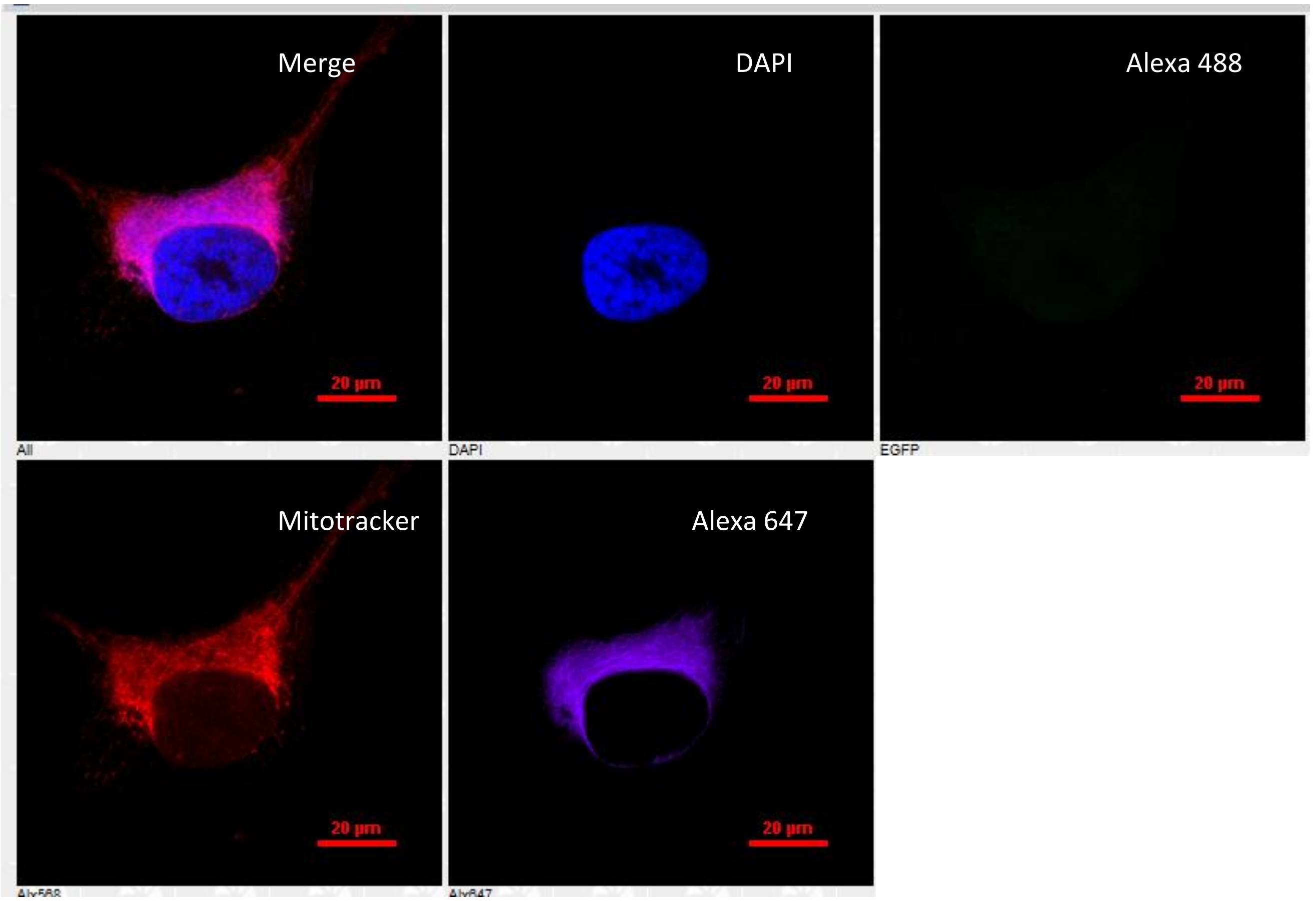

Supplement: File S1 — (PDF) [file pone.0098473.s001.pdf]
